# Exposure of the Swiss population to computed tomography

**DOI:** 10.1186/1471-2342-13-22

**Published:** 2013-07-30

**Authors:** Abbas Aroua, Eleni-Theano Samara, François O Bochud, Reto Meuli, Francis R Verdun

**Affiliations:** 1 Institute of Radiation Physics, Lausanne University Hospital, Lausanne, Switzerland; 2 Department of Diagnostic and Interventional Radiology, Lausanne University Hospital, Lausanne, Switzerland

**Keywords:** Diagnostic radiology, Computed tomography (CT), Medical radiation, X-rays, Population collective dose, Average effective dose

## Abstract

**Background:**

The frequency of CT procedures has registered a significant increase over the last decade, which led at the international level to an increasing concern on the radiological risk associated with the use of CT especially in paediatrics. This work aimed at investigating the use of computed tomography in Switzerland, following the evolution of CT frequency and dose data over a decade and comparing it to data reported in other countries.

**Methods:**

The frequency and dose data related to CT are obtained by means of a nationwide survey. National frequencies were established by projecting the collected data, using the ratio of the number of CT units belonging to the respondents to the total number of CT units in the country. The effective doses per examination were collected during an auditing campaign.

**Results:**

In 2008 about 0.8 Million CT procedures (~ 100 CT examinations / 1000 population) were performed in the country, leading to a collective effective dose of more than 6000 man.Sv (0.8 mSv/caput). In a decade the frequency of CT examinations averaged over the population and the associated average effective dose per caput increased by a factor of 2.2 and 2.9 respectively.

**Conclusions:**

Although the contribution of CT to the total medical X-rays is 6% in terms of the frequency, it represents 68% in terms of the collective effective dose. These results are comparable to those reported in a number of countries in Europe and America with similar health level.

## Background

Medical X-rays represent a major source of man-made irradiation of the population. In its 2010 Report, the United Nations Scientific Committee on the Effects of Atomic Radiation (UNSCEAR) indicated that although diagnostic radiology represents at the global level only 20% of the total annual per caput effective dose, it accounts for more than 94% of the man-made component [1]. In Switzerland diagnostic radiology was responsible in 2009 for 30% of the dose received by the population, but more that 92% of man-made irradiation [2]. This is why the exposure of the population by radiodiagnostics is periodically monitored (each 5–10 years) both at global and national level by UNSCEAR and national radiation protection authorities respectively. In Switzerland this monitoring has been guaranteed for decades by the Federal Office of Public Health (FOPH) and delegated to a research institute [3-6].

The frequency of computed tomography (CT) procedures has registered a significant increase over the last decade. This is due not only to evolution of the demographics and the ageing of the population, but may be attributed also to the technology advance in CT devices, particularly the introduction of multi-slice CT, which opened a new field of vascular investigations and led to the change of medical practice by often replacing fluoroscopy guided procedures with CT scans and to the proliferation of CT units in emergency departments.

CT is now contributing significantly to the total collective dose due to medical X-rays. UNSCEAR [1] estimates the CT dose contribution for developed countries (health level I) to 47% during the period of the study (1997–2007), while recent works reported in six European countries and the USA indicate CT dose contributions ranging from 46% up to 80% of the total collective dose for the years 2006 to 2008 [7-13]. In parallel to the widespread of CT, there has been an increasing concern at the international level on the radiological risk associated the use of this radiological modality especially in paediatrics reflected in scientific literature [14].

The aim of this work was to investigate the use of CT in Switzerland, to assess the evolution of this modality in terms of frequency of examinations and associated collective dose over the past decade and to compare the Swiss situation with other countries of similar health level.

## Materials and methods

All healthcare providers running an X-ray unit in Switzerland were addressed. This corresponds to 8,247 practices, radiology institutes and hospital departments, and 17,391 X-ray units authorized by the Regulatory Authority (FOPH). The participants were requested to provide their frequencies of examinations for the year 2008 in paper form by post mail, or in electronic form by email or by registration online. For this purpose, a dedicated website was developed (http://www.raddose.ch).

The Dose Datamed methodology explained in the European Guidance No 154 [15] was used. The X-ray examinations were grouped into seven radiological modalities: radiography, conventional fluoroscopy, diagnostic interventional radiology, therapeutic interventional radiology, computed tomography, dental radiology, mammography, and bone densitometry. Concerning CT, the participants were asked to give the annual number of procedures related to over 50 types, or in case this was not possible, for about 20 broader categories. The participants were also allowed to provide data in their own format. The data received in local categories were redistributed over the reference categories.

National frequencies were established by projecting the collected data to the whole population. The results corresponding to the participants in the survey were multiplied by the ratio of the number of CT units belonging to the participants to the total number of CT units in the country. The projection was performed separately for each category of healthcare provider (hospitals, practices, radiology institutes).

The effective doses associated to CT procedures were reviewed in an auditing campaign [16]. The tissue weighting factors (w_T_) provided by the International Commission on Radiological Protection in its Publication 60 [17] were used for the calculation of the effective dose. The effective dose was also calculated using w_T_ provided by ICRP Publication 103 [18], for the purpose of comparison. For CT modality, the new w_T_ resulted in a 2% increase of the collective dose. In the most recent investigation performed in the UK [9] a 2-3% decrease in the collective dose was registered for CT when using the new set of w_T_.

It is important to note here that for this survey no CT scans associated to SPECT/CT examinations or PET/CT examinations were taken into account, as they will be considered in a specific survey dedicated to the dose delivered in nuclear medicine [19].

## Results

The overall response rate was 45% in terms of number of X-ray units. It was 49% for radiology institutes and 63% for hospital departments.

Table 1 gives the frequency and dose data obtained in the survey concerning CT and all X-ray modalities. It shows the annual number of examinations performed in Switzerland in 2008 (7.7M population), the number per thousand population, as well as the associated annual collective dose and the average per caput effective dose. Over the 13 Million X-ray examinations performed 0.78 M are CT procedures, which corresponds to 6%. However, the CT contribution to the collective dose is revealed to be as high as 68%.

**Table 1:** 2008 Swiss annual frequency and dose data for CT and all X-ray modalities (rounded values)

	**CT**	**All X-ray modalities**
Number of examinations (in thousands)	780	13’000
Number of examinations/1000 population	100	1700
Collective dose (man.Sv)	6150	9100
Effective dose/caput (mSv)	0.8	1.2

Table 2 lists the most frequent and/or most irradiating CT procedures and presents the associated frequency and dose data. Full abdomen (upper and lower parts) is the most frequent (19% of total number of CT examinations) and most irradiating (29% of total CT dose) procedure. Chest procedure is the second most frequent (13%) and the third most irradiating (9%) procedure. Cerebrum procedure comes as the third most frequent (11%) and CT angiography as the second most irradiating (12%) procedures. For the latter procedure, although the frequency is low, the relatively high effective dose per procedure (up to 40 mSv in cardiac CT for example) leads to a high contribution to the collective dose.

**Table 2:** Frequency and dose data for the main CT examinations in terms of frequency and dose

	**Number of procedures/1000 population**	**Effective dose (mSv)/examination**	**Effective dose/caput (mSv)**
Full abdomen	19.7	11.7	0.230
Chest	13.1	5.4	0.708
Cerebrum	10.8	2.14	0.231
CT angiography	2.49	39	0.973

Table 3 shows the contribution of the different healthcare providers in terms of the frequency of CT procedures. Hospitals undertake 83.3% of the CT examinations, while radiology institutes perform 15.2% of the procedures. The remaining 1.5% is due to dental practices. Table 3 shows also that 11 big-size hospitals (university and canton) perform 44.7% while about 300 small-size hospitals and clinics (regional) perform 38.7 of the CT examinations. In terms of dose, hospitals are responsible of 83.5% and radiology institutes of 16.4% of the CT collective effective dose. The contribution of dental clinics is very low (0.1%).

**Table 3:** CT examinations performed in different healthcare providers in 2008

**Healthcare providers**	**N**	**Ratio**
	**(in thousands)**	**(%)**
University hospitals	211	27.1
Canton hospitals	137	17.6
Regional hospitals and clinics	301	38.7
Radiology institutes	118	15.2
Dental clinics	11.5	1.5

## Discussion

A study was conducted to assess the use of CT in Switzerland in the framework of a nationwide survey aiming at estimating the exposure of the population to medical X-rays. The 5–10 years periodic survey is a robust tool for radiation protection in medicine and the good response rates obtained guaranty statistically significant results.

Table 4 presents the number of all examinations and the number of CT examinations (/1000 population), the average effective dose due to radiodiagnostics and the average effective dose due to CT, for 1998 [4], 2003 [6] and 2008 [this work]. In one decade, the contribution of CT to the total medical X-rays increased from 3.4% to 6% in terms of the frequency and from 28% to 68% in terms of the collective effective dose.

**Table 4:** Comparison of 2008 frequency and dose data with previous surveys

**Year**	**Number of examinations/1000 population**		**N**_**CT **_**/N**_**Total**_	**E (mSv/caput)**		**E**_**CT**_**/E**_**Total**_
	**Total**	**CT**	**(%)**	**Total**	**CT**	**(%)**
1998	1340	46.2	3.4	1.0	0.28	28
2003	1470	76.7	5.2	1.2	0.56	47
2008	1680	100	6.0	1.2	0.80	68

Table 5 compares the CT frequency and dose data obtained in the present study with the data established in Switzerland in 1998 and 2003. Both the frequency of CT examinations and the associated collective effective dose registered a steady increase since 1998: respectively a factor of 2.2 and 2.9 in a decade. It should be noted that the increase was higher between 1998 and 2003 than between 2003 and 2008.

**Table 5:** 2008/1998 CT frequency and dose ratios

**Ratio**	**N/1000 population**	**E (mSv/caput)**
2003/1998	1.66	2.00
2008/2003	1.32	1.43
2008/1998	2.19	2.86

The increase in the number of CT examinations may be attributed partly to the 27% increase in the number of CT units in a decade (187 in 1998 and 238 in 2008), and partly to the technology advance in CT scanners that led to the change of medical practice with new indications for CT and the replacement of some fluoroscopy guided procedures with CT scans. In fact, the increase of the frequency of CT procedures is accompanied by a reduction in the number of diagnostic interventional procedures and radiography [20]. On the other hand the increase of the average effective dose per caput is due not only to the increase of the frequency of CT examinations, but also to the increase of the average effective dose per CT procedure.

Table 6 compares the number of CT examinations per 1000 population and the per caput average effective dose due to CT in Switzerland and in other countries of similar health level. The Swiss frequency (100/1000 population) and per caput average effective dose (0.8 mSv) are comparable to the French ones (115/1000 population, 0.8 mSv) and to those reported by UNSCEAR for countries of health level I (129/1000 population, 0.9 mSv). They lay in the range of frequencies (53-226/1000 population) and the range effective doses (0.3-1.5 mSv) reported in other countries. Table 6 reveals 3 categories of countries: low, medium and high consumers of CT. The first category is represented by the UK, the Netherland and Finland, the second category by France, Germany, Switzerland and to some extent Norway, and the third one by the USA. Table 6 shows also the contribution of CT examinations to the total number of examinations and to the collective effective dose in Switzerland with that reported in other countries. It shows clearly that the same pattern observed in Switzerland is registered elsewhere: a 10-20% contribution in terms of frequencies is reflected into up to a 2/3 contribution in terms of collective effective dose. In the case of Norway the CT frequency contribution is even higher (29%), since dental radiology is not considered.

**Table 6:** **Number of CT examinations (N**_**CT**_**) and effective dose due to CT (E**_**CT**_**) in various countries**

**Country**	**N**_**CT **_**/1000 population**	**% of total number of X-ray examinations**	**E**_**CT**_**/caput (mSv)**	**% of total effective dose**	**Reference**
Switzerland (2008)	100	6	0.8	68	This work
France (2007)*	115	10	0.8	65	[7]
Germany (2008)	132	8	1.0	60	[8]
United Kingdom (2008)	53	7	0.3	67	[9]
The Netherlands (2008)	62	11	0.4	46	[10]
Norway (2008)**	194	29	0.9	80	[11]
Finland (2008)	60	8.3	0.3	58	[12]
USA (2006)	226	18	1.5	66	[13]
UNSCEAR (1997–2007)	129	8	0.9	47	[1]

* Therapeutic interventional procedures excluded.

** Dental radiology is excluded in the total frequency.

## Conclusion

This investigation revealed that in 2008 the annual frequency of CT examinations performed in Switzerland was 0.78 Million, corresponding to 100 examinations per 1000 population. This is responsible for a collective effective dose of 6150 man.Sv or an average effective dose of 0.8 mSv/caput. From 1998 to 2008 the CT average frequency of examinations registered an increase of a factor 2.2 and the associated average effective dose increased by a factor 2.9. Computed tomography contributes 6% to the frequency of all medical X-rays and 68% to the total collective effective dose. This makes of CT the most irradiating radiological modality and the main contributor to the population dose due to radiodiagnostics, which is the case in other countries of similar health level. Compared to those countries, Switzerland appeared to be a medium consumer of CT and the efforts already engaged in radiation protection, notably the justification and optimisation of CT procedures should be maintained and consolidated; this is so important since an increase of the number of CT procedures is expected in the future due to the ageing of the population and the increase in healthcare needs.

## Keypoints

In 2008 about 0.8 Million computed tomography procedures (~ 100 CT examinations / 1000 population) were performed in Switzerland.

CT is the most irradiating radiological modality and the main contributor to the population dose due to radiodiagnostics.

Justification and optimisation of CT procedures should be maintained and consolidated.

## Competing interests

Financial competing interests

• In the past five years have you received reimbursements, fees, funding, or salary from an organization that may in any way gain or lose financially from the publication of this manuscript, either now or in the future? Is such an organization financing this manuscript (including the article-processing charge)? If so, please specify. **NO**

• Do you hold any stocks or shares in an organization that may in any way gain or lose financially from the publication of this manuscript, either now or in the future? If so, please specify. **NO**

• Do you hold or are you currently applying for any patents relating to the content of the manuscript? Have you received reimbursements, fees, funding, or salary from an organization that holds or has applied for patents relating to the content of the manuscript? If so, please specify. **NO**

• Do you have any other financial competing interests? If so, please specify. **NO**

Non-financial competing interests

• Are there any non-financial competing interests (political, personal, religious, ideological, academic, intellectual, commercial or any other) to declare in relation to this manuscript? If so, please specify. **NO**

The authors declare that they have no competing interests in submitting this paper.

## Authors’ contributions

AA and ETS contributed in the conception and design of the study, in the acquisition of data, in the analysis and interpretation of data as well as in drafting the manuscript. FOB, RM and FRV contributed in the conception and design of the study and in revising the manuscript critically. All authors read and approved the final manuscript.

## Pre-publication history

The pre-publication history for this paper can be accessed here:

http://www.biomedcentral.com/1471-2342/13/22/prepub
